# Prevalence of multidrug-resistant organisms in refugee patients, medical tourists and domestic patients admitted to a German university hospital

**DOI:** 10.1186/s12879-016-2105-y

**Published:** 2017-01-05

**Authors:** Claudia Reinheimer, Volkhard A. J. Kempf, Katalin Jozsa, Thomas A. Wichelhaus, Michael Hogardt, Fiona O’Rourke, Christian Brandt

**Affiliations:** Institute for Medical Microbiology and Infection Control, University Hospital Frankfurt, Paul Ehrlich-Straße 40, 60596 Frankfurt am Main, Germany

**Keywords:** Multidrug-resistant organisms, Refugees, Medical tourists, Infection control, Hospital hygiene

## Abstract

**Background:**

Patients with contact to healthcare-system in high-prevalence countries (HPC) and refugee patients in hospital settings (REF) have previously been identified to be at risk of carrying multidrug-resistant organisms (MDRO). Comparative studies addressing the epidemiology of MDRO in patients transferred from hospitals abroad (ABROAD) and REF are lacking but are necessary to introduce refined infection control measures.

**Methods:**

From December 2015 to June 2016, 117 REF, 84 ABROAD and 495 patients admitted to intensive care unit, with no refugee history or pre-treatment abroad (ICU), at University Hospital Frankfurt, Germany (UHF) were screened for MDRO on day of admittance. Data within these groups were compared and set in an epidemiological context.

**Results:**

52.1% (95% confidence interval = 42.7-61.5) of REF and 41.6% (31.0-52.9) of ABROAD, were positive for at least one MDRGN, respectively. In contrast, 7.9% (5.6-10.6) of ICU were positive for MDRGN. Thereof, 0.9% (0.0-4.7) of REF, 15.5% (8.5-25.0) of ABROAD and 0% (0.0-0.7) of ICU were positive for at least one MDRGN with carbapenem resistance (CR). In total, 19 MDRGN with CR were detected in ABROAD, with the most frequent species with CR being *A. baumannii* with 42.1% (20.3-66.5). Regarding MRSA, 10.3% (5.4-17.2) of REF, 5.9% (1.9-13.3) of ABROAD and a significantly lower proportion 1.4% (0.6-2.9) of ICU, respectively, were tested positive.

**Conclusions:**

Both REF and ABROAD pose a relevant hospital hygiene risk. High prevalence of MDRGN with CR in ABROAD was observed. Concise screening and infection control guidelines are needed in patient cohorts with increased risk for MDRO carriage.

## Background

Traveling to high-prevalence countries (HPC) for multidrug-resistant organisms (MDRO), such as multidrug-resistant *Enterobacteriaceae* and *Acinetobacter baumannii* (MDRGN) and methicillin-resistant *Staphylococcus aureus* (MRSA), as well as contact with the local healthcare-system in HPC have previously been identified as risk factors to for acquiring MDRO [1–9]. UHF is located in the direct vicinity of the Frankfurt international airport which processes almost 60 million passengers per year [10]. German residents returning after pre-treatment in healthcare-system in HPC, medical travelers seeking healthcare in Germany as well as travelers who experience medical problems during transit represent a relevant number of patients admitted to UHF.

Refugee patients’ countries of origin (COO) have previously been described as HPCs [11–13]. Since 2015, refugee influx to Germany has been high [12]. Concise hygiene management strategies therefore are required not only for refugees (REF) [11], but also for patients admitted from health-care systems abroad (ABROAD). Since the first study addressing the prevalence of multidrug-resistant organisms (MDRO) in refugee patients in hospital settings has been published in January 2016 [11], wide-ranging experiences in management of REF in European hospitals have been achieved. However, apart from a recent Dutch investigation [13], further data on MDRO in REF are available only to limited degree. This study therefore addresses the epidemiology of MDRO in refugee patients compared to patients after pre-treatment in hospitals abroad admitted to UHF between December 2015 and June 2016. Furthermore, these data were compared to the epidemiology of MDRO of residents, without any history of fleeing or pre-treatment abroad, within the same period. These data are needed to highlight challenges in terms of hospital hygiene and the need for specific demands on infection control and hospital hygiene.

## Methods

### Patients and specimens

We retrospectively evaluated data of 117 patients admitted from refugee accommodations (REF) to UHF between December 2015 and June 2016. All REF were systematically screened via rectal and nasal swabs for MDRO, i.e. MRSA and MDRGN. MDRGN are defined as *Enterobacteriaceae* with extended spectrum beta-lactamase (ESBL)–phenotype as well as *Enterobacteriaceae*, *Pseudomonas aeruginosa*, and *Acinetobacter baumannii* resistant against Piperacillin, any 3^rd^/4^th^ generation cephalosporin, and fluoroquinolones. MDRGN with additional resistance to carbapenems (CR) are assigned to “MDRGN with CR”. This approach is according to the hygiene plan of the University Hospital Frankfurt (UHF) and has previously been described [11]. In addition, 84 patients admitted from abroad (ABROAD), e.g. foreigners admitted to UHF for reasons of medical tourism or resident patients admitted to UHF for further treatment after initial treatment in foreign hospitals were screened for MDRO during the observational period. However, due to language difficulties in patient anamnesis, data concerning details (length of stay in hospitals abroad etc.) could not systematically be explored. We furthermore evaluated the MDRO prevalence among 495 German resident patients without refugee history or documented pre-treatment abroad, admitted to a intensive care unit (ICU) at UHF. Patients admitted for follow-up treatment from abroad or with refugee history were excluded from this ICU cohort.

We furthermore assume that vascular and thoracic surgery patients are more likely patients with long-term hospital history compared to traumatology patients (injured by e.g. road accidents). We therefore additionally investigated the MDRGN prevalence in surgical subgroup.

### Group ascertainment for REF and ABROAD

Subjects were fully assessed and assigned to groups according to several aspects. Distinguishability was covered by several aspects: first, we examined the patient data files for records of hospital stays abroad, residential status in a refugee accommodation, or records indicating a history of refugee status. We then investigated differences in funding of healthcare services also helped to distinguish patients into ABROAD and REF groups; German patients admitted from abroad are more likely to have health insurance whereas non-German patients from abroad (without refugee status) more commonly pay directly for services.

### Infection control measurements

According to German infection protection law (“*Infektionsschutzgesetz*”) it is mandatory for hospitals to execute a documented infection control strategy intended to prevent the transmission of infective agents and their potential harmful consequences on patients’ health. At UHF, all patients admitted from hospitals in HPC or arriving from refugee accommodations are pre-emptively isolated and screened for MDRO on day of admission. Screening procedure also applies to patients without history of pre-treatment abroad or refugee status admitted to an intensive care unit (ICU). Immediately after negative results for MDRGN and MRSA are available, patients are released from isolation. In case of a positive MDRGN and/or MRSA result, patients will remain in isolation during their entire stay at UHF to prevent MDRO transmission, as previously described [11].

### Detection of MDRGN and molecular resistance analysis

All laboratory testing was performed under strict quality-controlled criteria (laboratory accreditation according to ISO 15189:2007 standards; certificate number D–ML–13102–01–00, valid through January 25th, 2021). Rectal swabs were collected using culture swabs with Amies collection and transport medium (Hain Lifescience, Nehren, Germany) and streaked onto selective CHROMagarTM ESBL plates (Mast Diagnostica, Paris, France). Identification of presumed MDRGN species was done by matrix-assisted-laser desorption ionization-time of flight analysis (MALDI–TOF) and VITEK2 (bioMérieux, Nürtingen, Germany). Antibiotic susceptibility testing was performed according to Clinical and Laboratory Standards Institute (CLSI) guidelines using VITEK 2 and antibiotic gradient tests (bioMérieux). Carbapenemase encoding genes were detected via PCR analysis and subsequent sequencing from carbapenem-resistant *Enterobacteriaceae* including the *bla* genes for carbapenemases NDM, VIM, IMP, OXA–48, OXA–48 like and KPC as well as OXA–23, OXA–24, OXA–51, and OXA– 58 for *A. baumannii* [14–16].

### Detection of MRSA and determination of *spa* type

For the detection of MRSA, nasal swabs were inoculated on Brilliance MRSA Agar (Oxoid, Wesel, Germany). Identification of MRSA was done by MALDI–TOF and antibiotic susceptibility testing according to CLSI guidelines using VITEK 2. Clonal identity of MRSA isolates was analyzed by staphylococcal protein A (*spa*) typing using the Ridom StaphType software (Ridom GmbH, Würzburg, Germany) [17].

### Statistical analysis

Chi squared test was performed for statistical analysis. 95% confidence intervals (95% CI) for frequencies were calculated based on binomial distribution and used to confirm statistical significance. *P*-value calculations were not used to evaluate statistical significance as it has been criticized for low reliability [18].

## Results

Between December 2015 and June 2016, 117 REF and 84 ABROAD and 495 ICU were screened for MDRGN and MRSA by rectal and nasal swabs, respectively. The median age of REF, ABROAD and ICU was 19, 54 and 66 years, respectively. In the REF, the most frequently reported country of origin was Afghanistan (25.6%). The most frequently reported country ABROAD were admitted from was Turkey (13.1%). REF and ABROAD were most frequently admitted to the department of pediatrics (37.6%) and neurology (15.5%), respectively, as given in Table 1.

**Table 1:** Patient characteristics in REF, ABROAD and ICU

	REF	ABROAD	ICU
number of patients	117	84	495
mean age; standard deviation (years)	19; 14.7	54; 24.0	66; 14.4
male (n; %; 95%CI)	85; 72.7; 63.6-80.5	59; 70.2; 59.3-79.7	341; 68.9; 64.6-72.9
COO ^a^ / admitted from (n; %)			
Afghanistan	30; 25.6	1; 1.2	residents without refugee history or pre-treatment abroad
Syria	29; 24.8	-	
Somalia	8; 6.8	3; 3.6	
Algeria	7; 5.9	1; 1.2	
Iraq	5; 4.3	1; 1.2	
Pakistan	5; 4.3	-	
Eritrea	5; 4.3	2; 2.4	
Ethiopia	3; 2.6	3; 3.6	
Turkey	-	11; 13.1	
Greek	-	4; 4.8	
Italy	-	4; 4.8	
Morocco	-	4; 4.8	
Nigeria	-	4; 4.8	
India	-	3; 3.6	
Iran	-	3; 3.6	
Kuwait	-	3; 3.6	
Croatia	-	2; 2.4	
Ghana	-	2; 2.4	
Hungary	-	2; 2.4	
Saudi-Arabia	-	2; 2.4	
Spain	-	2; 2.4	
Sri Lanka	-	2; 2.4	
Thailand	-	2; 2.4	
Egypt	2; 1.7	4; 4.8	
Other	8; 6.8 ^b^	19; 22.6 ^c^	
Unknown	15; 12.8	-	
department patients admitted to (n; %; 95%CI)			
intensive care unit	-	-	495; 100
Surgery	13; 11.1; 0.6-18.3	12; 14.3; 7.6-23.6	495; 100; 99.3-100
general / vascular	6; 5.1 1.9-10.8	4; 4.7; 1.3-11.7	153; 30.9; 26.8-35.2
thoracic	-	2; 2.3; 0.2-8.3	273; 55.2; 50.7-59.6
traumatology	7; 5.9; 2.4-11.9	6; 7.1; 2.7-14.9	69; 13.9; 11.0-17.3
Internal medicine	20; 17.1; 10.8-25.2	39; 46.4; 35.5-57.6	
Infectious diseases	10; 8.5; 4.1-15.2	9; 10.7; 5.0-19.4	-
Gastroenterology	3; 2.6; 0.5-7.3	9; 10.7; 5.0-19.4	
Pneumology	2; 1.7; 0.2-6.0	5; 5.9; 1.9-13.3	
Hematology / Oncology	-	9; 10.7; 5.0-19.4	
Cardiology	3; 2.6; 0.5-7.3	7; 8.3; 3.4-16.4	
Angiology	1; 0.9; 0.0-4.7	-	
Rheumatology	1; 0.9; 0.0-4.7	-	
Gynecology / Obstetrics	10; 8.5; 4.1-15.2	3; 3.6; 0.7-10.1	-
Urology	6; 5.1; 1.9-10.8	1; 1.2; 0.0-6.5	
Pediatrics	44; 37.6; 28.8-47.0	8; 9.5; 4.2-17.9	
Neurology	4; 3.4; 0.9-8.5	13; 15.5; 8.5-25.0	
Psychiatrics	7; 5.9; 2.4-11.9	1; 1.2; 0.0-6.5	
other^d^	12; 10.3; 5.4-17.2	7; 8.3; 3.4-16.4	

(^a^) COO = country of origin; (^b^) = Albania, Iran, Jordan, Kosovo, Libya, Sudan, Tunisia, and Uganda with *n* = 1 each; (^c^) = Azerbaijan, Bangladesh, Bosnia, Bulgaria, Canada, China, Cuba, Cameroon, Denmark, Dominican Republic, Kazakhstan, Mozambique, Poland, Sudan, Taiwan, Togo, UK, USA, and Uzbekistan with *n* = 1 each; (^d^) = Ear-Nose-Throat (ENT), ophthalmology, orthopedics, and dermatology

The prevalence of at least one MDRGN in REF, ABROAD and ICU amounted to 52.1% (95% CI =42.7-61.5), 41.6% (31.0-52.9) and at a significantly lower prevalence 7.9% (5.6-10.6), respectively. Thereof, 0.9% (0.0-4.7) of REF, 15.5% (8.5-25.0) of ABROAD and 0% (0.0-0.7) of ICU, were positive for at least one MDRGN with additional carbapenem resistance (CR).

Six ABROAD were found positive for more than one MDRGN species with CR, resulting in a total number of 19 MDRGN with CR in ABROAD. Thereof, the most frequently detected MDRGN with CR in ABROAD was *A. baumannii* with 42.1% (20.3-66.5).

Interestingly, no significant difference was found in the overall prevalence of MDRGN between REF and ABROAD, however, the prevalence of MDRGN with CR in ABROAD (15.5%; 8.5-25.0) significantly exceeded the prevalence of MDRGN with CR in REF (0.9%; 0.0-4.7) by 17-fold (Table 2). The prevalence of at least one MDRGN with CR in both REF and ABROAD significantly exceeded the prevalence of MDRGN with CR in ICU (0%; 0.0-0.7). All carbapenemases detected are given in Table 2. Of the 117 REF tested, 61 were tested positive for at least one MDRGN, three individuals were found positive for two different MDRGN each and one individual was found positive for three different MDRGN, resulting in a total number of 66 MDRGN in REF. Of the 84 ABROAD thested, 35 patients were tested positive for at least one MDRGN, three individuals were positive for two different MDRGN each, two individuals were positive for three MDRGN each, and two individuals were positive for four different MDRGN each, resulting in a total number of 48 MDRGN in ABROAD. Of note, two individuals were tested positive for three different MDRGN with CR each: *n* = 1 with *A. baumannii, K. pneumoniae* and *E. coli,* and *n* = 1 with *A. baumannii, K. pneumoniae* and *Proteus mirabilis*. In contrast, all of 495 ICU were tested single-positive for MDRGN, resulting in 39 MDRGN in this group.

**Table 2:** Microbiological findings in REF, ABROAD and ICU

	REF	ABROAD	ICU
number of patients	117	84	495
patients positive for at least one MDRGN (n;%; 95% CI)	61; 52.1; 42.7-61.5	35; 41.6; 31.0-52.9	39; 7.9; 5.6-10.6
subgroup surgery			
general / vascular	4; 66.7; 22.2-96.6	1; 25; 0.6-80.6	22; 14.4; 9.2-20.9
thoracic	-	1; 50; 12.6-98.7	14; 5.1; 2.8-8.5
traumatology	3; 42.9; 9.9-81.6	2; 33.3; 4.3-77.7	3; 4.3; 0.9-12.2
patients positive for at least one MDRGN with CR (n;%; 95%CI)	1; 0.9; 0.0-4.7	13; 15.5; 8.5-25.0	0; 0; 0.0-0.7
total number of MDRGN	66	48	40
total number of MDRGN with CR	1	19	-
*Escherichia coli*			
ESBL (n;%; 95CI)	24; 20.5; 13.6-28.9	5; 5.9; 1.9-13.3	14; 2.8; 1.5-4.7
ESBL/FQ (n;%;95CI)	34; 29.1; 21.0-38.2	17; 20.2; 12.3-30.4	19; 3.8; 2.3-5.9
ESBL/FQ + CR (n;%;95CI)	1; 0.9; 0.0-4.7	2; 2.4; 0.3-8.3	-
carbapenemases (n)	none detected (1)	OXA-181 + NDM-5 (1) none detected (1)	-
*Klebsiella pneumoniae*			
ESBL (n;%;95CI)	3; 2.6; 0.5-7.3	-	1; 0.2; 0.0-1.1
ESBL/FQ (n;%;95CI)	4; 3.4; 0.9-8.5	5; 5.9; 1.9-13.3	2; 0.4; 0.0-1.5
ESBL/FQ + CR (n;%;95CI)	-	7; 8.3; 3.4-16.4	-
carbapenemases (n)	-	OXA-48 (2) OXA-181 (1) KPC-3 (1) KPC-9 (1) NDM-1 (1) none detected (1)	-
other *Enterobacteriaceae*			
Ceph/FQ (n;%;95CI)	-	2 ^a^; 2.4; 0.3-8.3	4 ^b^; 0.8; 0.2-2.1
Ceph/FQ + CR (n;%;95CI)	-	1 ^c^; 1.2; 0.0-6.5	-
carbapenemase (n)	-	none detected (1)	-
*Acinetobacter baumannii*			
Ceph/FQ (n;%;95CI) Ceph/FQ + CR (n;%;95CI)	-	-	-
	-	8; 9.5; 4.2-17.9	-
carbapenemase (n) ^d^	-	OXA-23 (4) OXA-24 (1) OXA-58 (1) NDM-1 + OXA-23 (1) OXA-23 + OXA-24 (1)	-
*Pseudomonas aeruginosa*			
Pip/Ceph/FQ + CR (n;%;95CI)	-	1; 1.2; 0.0-6.5	-
carbapenemase (n)	-	none detected (1)	-
patients positive for MRSA (n;%; 95% CI)	12; 10.3; 5.4-17.2	4; 4.8; 1.3-11.7	7; 1.4; 0.6-2.9
*spa* types (n)	t304 (2) t4892 (2)		
	t044 (1) t131 (1) t311 (1) t325 (1) t386 (1)	t003 (1) t217 (1) t325 (1) t608 (1)	t020 (2) t003 (1) t223 (1) t309 (1) t668 (1) t3758 (1)
	t688 (1) t790 (1) t3422 (1)		

*Abbreviations*: *CR* carbapenem resistance, *ESBL* extended spectrum beta-lactamase, *ESBL/FQ* ESBL and additional resistance to fluoroquinolones, *Ceph/FQ* resistance to cephalosporins and additionally to fluoroquinolones, *Pip/Ceph/FQ + CR* resistance to Piperacillin and additionally Ceph/FQ + CR, *MRSA* methicillin-resistant *Staphylococcus aureus*

(^a^) = *Morganella morganii*, *Proteus mirabilis* (*n* = 1 each); (^b^) = *Enterobacter cloacae*, *Serratia marcescens*, *Proteus mirabilis*, and *Morganella morganii* (*n* = 1 each); (^c^) = *Proteus mirabilis*; (^d^) = other than species-specific OXA-51

The most common MDRGN in REF, ABROAD and ICU was *E. coli* expressing resistance due to ESBL and additional resistance to fluoroquinolones (Table 2), with 34/117 (29.1%; 21.0-38.2) in REF, 17/84 (20.2%; 12.3-30.4) in ABROAD, and, significantly lower, 19/495 (3.8%; 2.3-5.9) in ICU (Table 2). Furthermore, 10.3% of samples (5.4-17.2) in REF and 4.8% of samples (1.3-11.7) in ABROAD, respectively, were positive for MRSA. The prevalence of MRSA in ICU (1.4%; 0.6-2.9) was significantly lower compared to REF. *Spa* types detected in REF, ABROAD and ICU are given in Table 2.

Furthermore, we found a low prevalence of MDRGN in the REF, ABROAD and ICU surgical group (Table 2). In particular, in the traumatology ICU group, the prevalence of MDRGN was lowest with 4.3% (0.9-12.2).

## Discussion

The rapid global spread of MDRO is a serious global health risk and must direct attention to the development of effective strategies to prevent the spread of antibiotic resistances and life-threatening infections caused by MDRO. Diligent prevention strategies of MDRO transmission in hospital settings should therefore be the focus of preventive efforts. Traveling to HPC, medical tourism, contact to local health care systems as well as history of refugee status have previously been identified as risk factors for carrying MDRO [1–11, 19, 20].

The objective of our study was to give a firm insight into the epidemiology of MDRO in refugee patients (REF), patients admitted from abroad (ABROAD) and German resident patients admitted to a intensive care unit at UHF (ICU). The major strength of our investigation is the direct comparison of these three groups as this issue for our knowledge has not been addressed in scientific literature so far.

No significant difference in the overall prevalence of MDRGN between REF and ABROAD was found, the prevalence of MDRGN with CR in ABROAD (15.5%), however, significantly exceeded the prevalence in REF (0.9%) by 17-fold (Table 2). This phenomenon might reflect an inherent risk to acquire highly resistant MDRGN in hospitals in HPC, from which the majority of ABROAD are admitted from to UHF, such as e.g. Turkey, Egypt, Ethiopia or Somalia (Table 1). For repatriates, this aspect has previously also been mentioned by *Josseaume* et al. [8]. Considering that conditions under which refugees make their way to Germany might have been poor, we hypothesize that refugees in good health condition are more likely to make the journey than refugees suffering from severe disease or disability and with a strong history of hospital treatment in their country of origin. We however cannot quantify the level of contact REF have had with their local health care system, which therefore might be a source of bias in our setting. In a future setting, it therefore might be interesting to evaluate the MDRO prevalence in refugees without any contact to their local health care system compared to refugees without.

Our data thus indicate, that ABROAD are more likely to represent the prevalence of highly resistant bacteria in hospitals abroad, whereas REF are more likely to portray the general prevalence of highly resistant bacteria in their COO. Furthermore, this finding is additionally underlined by previous findings showing a high prevalence of *A. baumannii* with CR in hospitals in Syria and Iran [21–24].

However, due to language barriers, data regarding e.g. exact previous medical treatment or length of stay in the hospitals abroad was available only in a minority of REF patients as well as a low number of ABROAD patients. This aspect could be interesting to evaluate the timeframe that MDRO might be acquired within in hospitals abroad. Moreover, exact phylogeny analysis of isolated resistant strains (e.g., via NGS techniques) was not performed which might have demonstrated a potential clonal spread of resistant pathogens in some particular countries of patients from ABROAD.

In the traumatology ICU group we found a MDRGN prevalence of 4.3% (0.9-12.2), which is in the range of general German population’s MDRGN prevalence [25].

Concerning prevalence of MRSA, no significant difference between REF (10.3%; 5.4-17.2) and ABROAD (4.8%; 1.3-11.7) was observed, even though MRSA prevalence in ICU (1.4%; 0.6-2.9) was significantly lower than in REF (Table 2). This finding might reflect the limited space conditions in refugee accommodations: in parallel with e.g. an outbreak of measles in a refugee settlement in Calais, France, [26], or clustering of shigellosis in refugees in Austria [27]. The conditions refugees live in might also be conducive to the spread of MRSA within the community. Investigating the *spa* types in the three cohorts, we found ICU patients harbouring *spa* types known to be common in Germany, e.g. t003, t020, or t223 [28, 29]. In contrast, many of the *spa* types detected in REF (t131, t304, t325, t790, t4892; Table 2) have frequently been recorded from e.g. Iran, Jordan, United Arab Emirates or Lebanon [28]. However, the number of MRSA detected in REF in this study (*n* = 12) seems to be too low to assess any epidemiological trends.

These mentioned countries are in immediate neighbourhood to the most frequently recorded COO of REF in this study (e.g. Afghanistan and Syria, Table 1), which might indicate that REF bring new *spa* types to Germany and Europe. For future settings, we suggest to perform genotyping on the core chromosomes, e.g. via multilocus sequence typing (MLST), whole genome sequencing or SCCmec typing for MRSA, and plasmid typing for more exact phylogenetic and epidemiological investigations.

## Conclusions

In summary, our findings demonstrate a significant and inherent risk for REF and ABROAD to carry MDRGN and MRSA. While it is hardly possible to predict whether these MDRGN and MRSA strains will have evolutionary advantage in German population or in German hospitals, this investigation revealed the indispensability of screening programs and appropriate hygiene measurements in refugee patients as well as in patients pre-treated in hospitals abroad. In particular, the overwhelming high proportion of MDRGN with CR in patients after pre-treatment in hospitals abroad should be considered by domestic healthcare systems. Therefore, this risk must be covered by adequate hospital infection control measurements. Based on our findings, we feel a strong need to implement a concise screening procedure for patients arriving from abroad as well as refugee patients. This should also include German residents after pre-treatment in hospitals abroad. We therefore suggest screening for MDRO on day of admittance as well as pre-emptive isolation for both groups.

## Abbreviations

95%CI: 95% confidence interval
ABROAD: Patients with pre-treatment in abroad admitted to UHF
CLSI: Clinical and Laboratory Standards Institute
COO: Country of origin
CR: Carbapenem resistance
ENT: Ear-nose-throat
ESBL: Extended spectrum beta-lactamase
HPC: High-prevalence country
ICU: Patients admitted to intensive care unit (without refugee history nor pre-treatment abroad)
MDRGN: Multidrug-resistant gram-negative bacteria
MDRO: Multidrug-resistant organisms
MLST: Multilocus sequence typing
MRSA: Methicillin-resistant *Staphylococcus aureus*
PCR: Polymerase chain reaction
REF: Refugee patients
UHF: University Hospital Frankfurt, Germany
UK: United Kingdom
USA: United States of America
